# Insulin-degrading enzyme prevents α-synuclein fibril formation in a nonproteolytical manner

**DOI:** 10.1038/srep12531

**Published:** 2015-07-31

**Authors:** Sandeep K. Sharma, Erik Chorell, Pär Steneberg, Emma Vernersson-Lindahl, Helena Edlund, Pernilla Wittung-Stafshede

**Affiliations:** 1 Department of Chemistry, Umeå University, 90187 Umeå, Sweden; 2 Umeå Center for Molecular Medicine, Umeå University, 90187 Umeå, Sweden

## Abstract

The insulin-degrading enzyme (IDE) degrades amyloidogenic proteins such as Amyloid β (Αβ) and Islet Amyloid Polypeptide (IAPP), i.e. peptides associated with Alzheimer’s disease and type 2 diabetes, respectively. In addition to the protease activity normally associated with IDE function an additional activity involving the formation of stable, irreversible complexes with both Αβ and α-synuclein, an amyloidogenic protein involved in Parkinson’s disease, was recently proposed. Here, we have investigated the functional consequences of IDE-α-synuclein interactions *in vitro*. We demonstrate that IDE in a nonproteolytic manner and at sub-stoichiometric ratios efficiently inhibits α-synuclein fibril formation by binding to α-synuclein oligomers making them inert to amyloid formation. Moreover, we show that, within a defined range of α-synuclein concentrations, interaction with α-synuclein oligomers increases IDE’s proteolytic activity on a fluorogenic substrate. We propose that the outcomes of IDE-α-synuclein interactions, i.e. protection against α-synuclein amyloid formation and stimulated IDE protease activity, may be protective *in vivo*.

Accumulating data suggest that type 2 diabetes (T2D) and Alzheimer’s disease (AD) are associated and both diseases share the pathological characteristic of amyloid deposits, derived primarily from Islet Amyloid Polypeptide (IAPP) in T2D β-cells and Amyloid β (Αβ) in AD neurons^1^,^2^,^3^,^4^. Similarly, several independent reports suggest that T2D patients are predisposed towards developing Parkinson’s Disease (PD), in which aggregation of α-synuclein into amyloid fibers is believed to be instrumental in the toxic process that cause degeneration of dopaminergic neurons in PD patients^5^,^6^. Insulin-degrading enzyme (IDE) is a highly conserved Zn metallopeptidase involved in the degradation of insulin and a variety of other small peptides that can form β-pleated sheet-rich fibrils including IAPP, Aβ, bradykinin and somatostatin^7^,^8^. Genome-wide association studies have linked *IDE* to both T2D and AD^9^,^10^ and *Ide* mutant mice have increased levels of IAPP, insulin, and α-synuclein in pancreatic islets as well as increased cerebral levels of Αβ^11^,^12^. Thus, IDE appears to exert a key role in the turnover of amyloidogenic proteins and consequently in preventing toxic amyloid formation. In addition to the proteolytical role for IDE in neutralizing amyloidogenic peptides an unexpected property to form a stable complex with both Αβ and α-synuclein was recently shown *in vitro* and a protective ‘dead-end chaperone’ function was proposed for IDE^11^,^13^,^14^.

*Ide* knock out (KO) mice have impaired β-cell function including impaired glucose-stimulated insulin secretion and reduced islet autophagic flux and microtubule content^11^. Additionally, IDE and α-synuclein levels were found to be inversely correlated in β-cells of *Ide* KO mice and T2D patients^11^. α-synuclein is a 140-residue synaptic signaling protein normally associated with PD^5^,^6^,^15^. It consists of three parts, the N-terminus (residues 1–60) that can interact with membranes, the amyloidogenic so-called NAC domain (residues 61–95), and the acidic C-terminus (residues 96–140). Although considered an intrinsically unstructured polypeptide, internal auto-inhibitory interactions have been reported; deletion of either the N- or C-terminal part of α-synuclein results in faster amyloid formation^16^,^17^. With its 140 residues, α-synuclein is likely not a substrate for IDE degradation as it has been shown that IDE can only cleave substrate peptides of 50 amino acids or shorter^18^. Nonetheless, we recently showed that, similar to what has been reported for Aβ^13^,^14^, IDE formed SDS resistant complexes with α-synuclein *in vitro*^11^.

IDE exists in solution as a mixture of monomers and dimers; it is unique among M16 metallopeptidases in that it exhibits allosteric kinetic behavior^19^,^20^. Crystal structures have revealed that IDE consists of two structurally related domains (IDE-N; IDE-C) that adopt a clamshell-like structure enclosing a large central chamber^18^,^21^,^22^. IDE-N harbors an intact active site characteristic of Zn metallopeptidases. Substrate binding requires a conformational change that opens the central chamber and enables electrostatic interactions inside the chamber. In addition to interactions with the catalytic site, large peptides can interact with a distal, positively charged binding site located in IDE-C, which is located 30 Å away from the catalytic site^23^ and plays a key role in allosteric and heterotropic activation^18^. The rate-limiting step for IDE proteolytical activity appears to be the conformational switch from closed to open state^18^,^21^,^22^.

Here we have characterized the consequences of IDE-α-synuclein interactions *in vitro* and show that IDE, in a nonproteolytical manner, efficiently inhibits α-synuclein amyloid formation and, instead, smaller α-synuclein oligomers accumulate. Intriguingly, we also found that IDE interaction with α-synuclein oligomers, in a concentration dependent manner, enhance the proteolytical activity of IDE on a fluorogenic substrate.

## Results

### IDE inhibits α-synuclein amyloid formation

**Figure 1:**
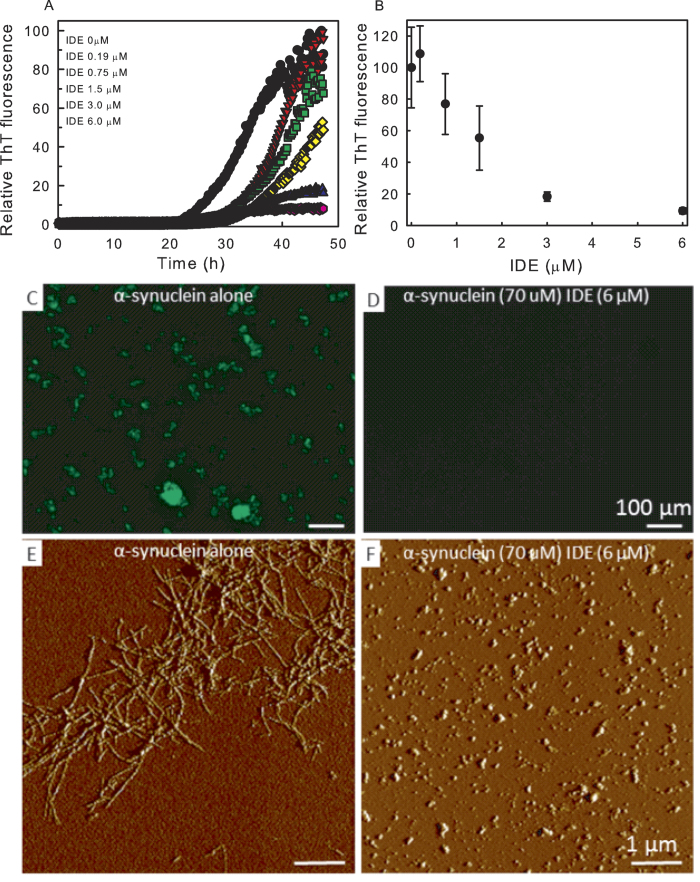
Effect of IDE on α-synuclein amyloid formation. (**A**) ThT fluorescence versus time (37 °C, agitation) probing amyloid formation of 70 μM α-synuclein without and with the presence of various concentrations of IDE between 0.2 and 6.0 μM. (**B**) Normalized ThT emission after 48 h of incubation (endpoint of experiments) for α-synuclein in the presence of varying concentrations of IDE. Error bars refer to triplicate experiments. Fluorescence microscopy of α-synuclein samples after ThT assays without (**C**) and with (**D**) the presence of 6 μM IDE (excitation of ThT). Scale bar 100 μm. AFM images of α-synuclein after ThT assays without (**E**) and with (**F**) the presence of 6 μM IDE. Scale bar 1 μm.

To analyze the effect of IDE on α-synuclein aggregation we first probed α-synuclein fibril formation using a thioflavin T (ThT) fluorescence assay^24^. In aggregation assays using α-synuclein alone at a concentration of 70 μM (37 °C, agitation), a lag phase of about 20 hrs was observed after which the ThT emission rapidly increased, indicative of amyloid fiber formation (Fig. 1A). Under the same conditions, addition of IDE efficiently blocked the formation of α-synuclein amyloid fibers, as evident from IDE-concentration dependent decreases in ThT emission (Fig. 1A). The inhibitory effect of IDE on α-synuclein aggregation exhibited a half maximal effect at 1:40 molar ratio IDE to α-synuclein (Fig. 1B). Control experiments demonstrated that IDE itself did not interact with ThT (Figure S1A). The inhibition of α-synuclein amyloid fiber formation by IDE was confirmed using fluorescence microscopy, which showed that the large fluorescent aggregates of fibers observed for α-synuclein incubated alone, were absent in the presence of IDE (Fig. 1C,D). Atomic Force Microscopy (AFM) analyses demonstrated that in contrast to the distinct amyloid fibers formed with α-synuclein alone, the addition of IDE to α-synuclein prevented the formation of such fibers and instead smaller, oligomeric α-synuclein species were observed (Fig. 1E,F). Given that IDE does not degrade α-synuclein even if incubated up to 48 h (Figure S1B and^11^), these data provide evidence that non-proteolytical interactions between IDE and α-synuclein prevents α-synuclein fibril formation.

As a control, we added an unrelated large protein, bovine serum albumin (BSA), to fresh α-synuclein and found that it failed to block α-synuclein aggregation as probed by ThT (Figure S2A). We also tested whether addition of IDE to preformed α-synuclein fibers could dissolve such aggregates. IDE was however not able to decrease the ThT emission from preformed α-synuclein fibers (Figure S2B). Taken together these data show that IDE, in a concentration-dependent manner and at sub-stoichiometric ratios, antagonizes α-synuclein amyloid fiber formation. The inhibition of α-synuclein amyloid formation found for IDE were not affected by the inclusion of ATP in the mixtures (Figure S3).

**Figure 2:**
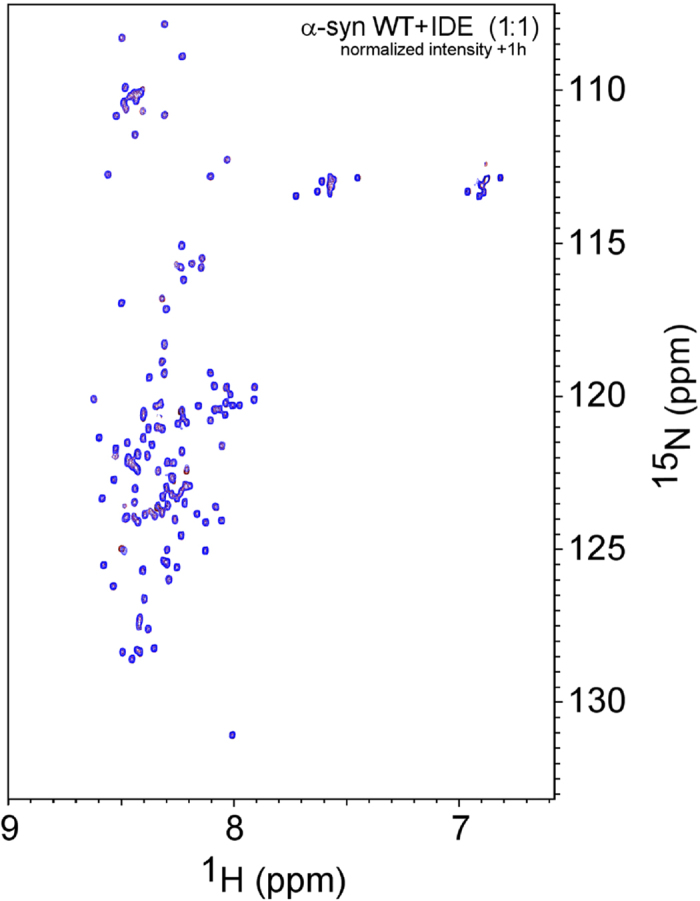
No IDE interaction with monomeric α-synuclein. ^1^H-^15^N HSQC spectra of ^15^N-labeled α-synuclein alone (red) and 1 h after the addition of IDE (1:1 molar ratio; blue) in phosphate buffer, pH 7.4, 10 °C. Only very small shifts of a few cross peaks are detected upon the addition of IDE, indicating at the most a transient interaction between IDE and α-synuclien monomers.

Isothermal titration calorimetry (ITC) experiments indicated that there was no enthalpic interaction between IDE and α-synuclein monomers at neither 20 nor 30 °C. In contrast, when IDE was titrated to pre-formed α-synuclein oligomers, a cooperative transition, with negative enthalpy change, was detected at both temperatures (Figure S4). The stoichiometry of the interaction indicated binding of two-three IDE molecules per α-synuclein oligomer assuming, about 15–20 α-synuclein monomers per oligomer^25^. In accord with the ITC data, when mixing ^15^N-labeled α-synuclein monomers with unlabeled IDE at a 1:1 molar ratio, the solution NMR ^1^H-^15^N HSQC spectrum did not present any evidence for an interaction between the two proteins (Fig. 2). These results suggest that, similar to what has been observed for DnaK interactions with α-synuclein^26^, IDE interacts with α-synuclein oligomers but stable interactions with monomers are not favored.

### Characterization of IDE-induced α-synuclein oligomers

Size-exclusion chromatography (SEC) analysis of an incubated sample of IDE and α-synuclein (37 °C, 48 h, agitation) revealed an oligomer peak that was further analyzed by SDS-PAGE (Figure S5A). We have earlier reported that α-synuclein oligomers are not stable towards SEC and most of the protein ends up in the monomer elution peak^27^ which also appeared to be the case for the IDE-α-synuclein oligomers (Figure S5A). The SDS-PAGE data of the oligomer fraction revealed the previously reported IDE-α-synuclein 1:1 complex^11^, supporting that IDE is bound the α-synuclein oligomers that are loaded on the column (as expected based on the ITC data). Far-UV circular dichroism analysis of the IDE-α-synuclein oligomers revealed β-sheet content for α-synuclein in the oligomers (Figure S5B). This is similar to what we reported for the FN075-triggered α-synuclein oligomers^27^ and imply that upon IDE binding to these oligomers, reported to be on-path-like, further aggregation is arrested. From DLS analysis of the IDE-α-synuclein oligomer samples, an estimate of the size distribution could be determined. IDE-α-synuclein oligomers have a hydrodynamic radius centered around ~ 35 nm, which is larger than the FN075-α-synuclein oligomer^27^, in accord with IDE, being a large protein, increasing the overall dimensions (Figure S5C).

### IDE-α-synuclein interactions stimulates IDE proteolytic activity

**Figure 3:**
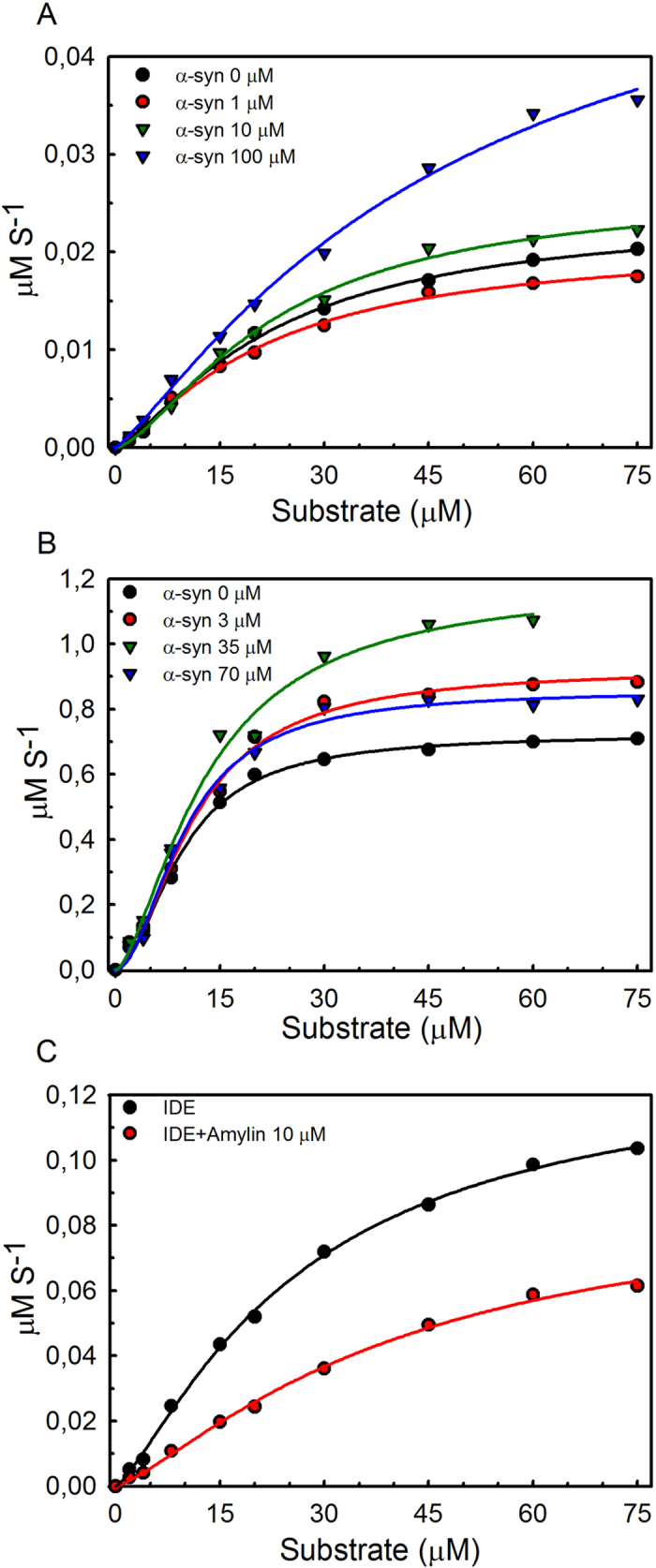
Effect of α-synuclein on IDE proteolytical activity. Activity data (initial rates versus substrate concentration) for IDE proteolysis of fluorogenic substrate V at 37 °C (**A**, 1 μM IDE; **B**, 3 μM IDE) as a function of α-synuclein concentration. Samples of IDE and various concentrations of α-synuclein were incubated for 48 hours at 37 °C prior to the activity analysis. (**C**) Activity data (initial rates versus substrate concentration) for 1.6 μM IDE proteolysis of fluorogenic substrate V at 37 °C without and with the presence of 10 μM amylin (competing substrate). Enzymatic parameters from fits to a sigmoidal equation are summarized in Table 1.

To assess the consequence of IDE-α-synuclein interactions on IDE’s proteolytic function, we probed the IDE proteolytic activity using a fluorogenic substrate (substrate V) in the presence of increasing amounts of α-synuclein. Substrate V is an internally quenched fluorescent peptide derived from the amino acid sequence of bradykinin^28^. IDE-α-synuclein samples, at indicated concentrations, were pre-incubated at the same conditions at which α-synuclein amyloid formation was inhibited by IDE (i.e., 48 h incubation at 37 °C with agitation) prior to the addition of substrate V. In agreement with previous data^20^, we observed that the data for IDE catalytic activity (initial rates versus substrate concentration) involved cooperativity and sigmoidal fits were used to extract enzyme activity parameters (Table 1). Intriguingly, we find that the proteolytic activity of IDE on substrate V, both in terms of turn-over number (k_cat_) and catalytic efficiency (k_cat_/K_M_), was increased in the presence of α-synuclein (Fig. 3A,B).

We noted that IDE itself was about 25-fold more active in terms of both k_cat_ and k_cat_/K_M_ at 3 μM than at 1 μM (Table 1). In addition, the cooperativity number n is increased (from 1.3 to 1.9) at the higher IDE concentration. This parameter has been linked to allostery in IDE dimers^29^. Given that IDE dimers have been suggested to be much more active than monomers^29^, we suspected that the differential data reflected that there was more dimers in the 3 μM samples than in the 1 μM samples. To test this hypothesis, we used size-exclusion chromatography as a method to probe the IDE assembly status at different concentrations. SEC data for three different IDE concentrations (all resulting in elution peak concentrations in the low μM range) revealed a concentration-dependent distribution between monomers and dimers. For the lowest IDE concentration loaded on the column, IDE eluted as a monomer (1 μM concentration in this elution peak) whereas for the highest IDE concentration loaded, IDE eluted as mostly dimers (3 μM concentration in this elution peak). For an intermediate IDE concentration loaded on the column, a mixture of monomers and dimers was observed (Figure S6). Thus, the IDE monomer-dimer dissociation constant appears to be in the low μM range. As a consequence, IDE monomers will dominate at the low concentration (1 μM) and IDE dimers will dominate at the high concentration (3 μM), providing an explanation for its apparent increased intrinsic activity (Table 1).

The enhancement of IDE’s proteolytical activity by α-synuclein increased linearly with α-synuclein concentration for the 1 μM IDE condition (Table 1, Figure S7): the more α-synuclein present, the higher the IDE activity. In contrast, the correlation between α-synuclein concentration and IDE activity effect appeared to be nonlinear for higher IDE concentrations; 70 μM α-synuclein was less effective than 35 μM α-synuclein in enhancing the proteolytical activity of 3 μM IDE (Table 1, Fig. 3B and S7). This suggests that at increased α-synuclein levels, when the IDE levels are sufficiently high, several IDE molecules bind to the same α-synuclein oligomer obscuring binding of proteolytical substrates due to local crowding.

As expected the addition of IAPP, which in contrast to α-synuclein is degraded by IDE, decreased the protelytical degradation of the fluorogenic substrate. This is in agreement with substrate competition for IDE’s proteolytical activity (Fig. 3C). As an additional control, we probed IDE activity without and with the presence of ThT to assure that ThT did not interact with IDE in a non-fluorescent manner. We found no difference upon addition of ThT, confirming that ThT has no effect on IDE proteolytic activity (Figure S7).

## Discussion

A key protective cellular role for IDE is to limit intracellular levels of aggregate-prone, amyloidogenic peptides such as IAPP and Aβ, by ensuring their degradation^7^,^8^. Recently an additional mechanism by which IDE neutralizes amyloidogenic peptides was proposed that was based on the observation that IDE could form complexes with both Aβ and α-synuclein without degradation^11^,^13^,^14^. Here we have investigated the consequences of this so called ‘dead-end-chaperone’ activity of IDE on α-synuclein amyloid fibril formation *in vitro*. Our data show that IDE efficiently inhibits α-synuclein amyloid formation *in vitro* and instead α−synuclein oligomers accumulate. Our biophysical data suggest that IDE does not interact with α−synuclein monomers but instead binds to α−synuclein oligomers that form during the aggregation process, blocking them from further assembly into amyloid fibers. This mechanism is similar to the one proposed for human serum albumin and transferrin inhibition of Aβ amyloid formation, systems that have been characterized in great detail^30^,^31^,^32^,^33^,^34^,^35^. Concurrent with amyloid inhibition, we found that the α-synuclein interactions enhanced the proteolytical activity of IDE on small substrates. Intriguingly, the positive effect of α-synuclein on IDE’s proteolytical activity was reduced at high IDE concentrations, leaving open the possibility that high levels of α-synuclein not only may lead to enhanced risk of α-synuclein aggregation itself, but also to a decrease in IDE-mediated degradation of amyloidogenic substrates such as IAPP and Aβ.

In sharp contrast to the Aβ complex with IDE, which was proposed to involve inactive species of IDE^36^, we provide evidence that the complex between IDE and α-synuclein results in activation of IDE’s proteolytic activity on small substrates. This difference may be explained by binding of Aβ to the active site of inactive IDE molecules that, due to their inactivity, does not cleave the Aβ peptides. In contrast, we propose that α-synuclein binds to the exosite in IDE that, when filled, is known to allosterically activate cleavage^23^.

Molecular-chemical inspection of the α-synuclein sequence and IDE chamber electrostatics suggests that residues 100–140 of the highly acidic C-terminal part of α-synuclein will favor binding to the positively charged exosite region in IDE. Moreover, since the C-terminal 40 residues are exposed in α-synuclein oligomers^37^, this notion is compatible with the observation of α-synuclein oligomers in the presence of IDE in aggregation assays (Fig. 1) as well as the direct binding of IDE to pre-formed α-synuclein oligomers (Figure S4). Because the C- and the N-termini of α−synuclein are known to engage in auto-inhibitory interactions in the monomer state^16^,^17^, the C-termini may be more exposed in oligomers than in monomers. The complete α-synuclein polypeptide is too large to fit in the IDE chamber^18^; instead we propose that the C-terminus binds in the chamber whereas the rest of the polypeptide remains outside. This has two implications: first, residues 1–100 of α-synuclein can retain interactions with other α-synuclein molecules in the oligomer and, second, the IDE chamber may be forced into an open conformation. Since the conformational switch between closed and open states is rate limiting for IDE’s proteolytical activity^18^,^21^,^22^, binding of a molecule that favors the open conformation is likely to enhance substrate binding and product release. An additional mechanism that may be relevant for the effect of IDE-α-synuclein interactions on IDE’s proteolytical activity is steric effects; if α-synuclein fills the exosite, catalytically nonproductive substrate binding is blocked.

T2D is associated with both age and obesity and IDE activity has been shown to decline with age^38^. We have previously shown that reduced IDE activity in mice results in impaired autophagy and consequently decreased turnover and/or neutralization of amylidogenic proteins in β-cells^11^. In dopaminergic neurons of the substantia nigra, the assembly of α-synuclein into toxic species, which triggers neurodegeneration and PD, is also likely increased with age. Our findings demonstrate that there is a direct link between α-synuclein and IDE *in vitro*. We found that α-synuclein amyloid formation is efficiently blocked by IDE *in vitro*, which may be protective towards initiation of PD in dopaminergic neurons, and possibly also against α-synuclein fibril formation in AD neurons and T2D β-cells. The internal NAC region of α-synuclein has been isolated from AD amyloid deposits *in vivo* and both the NAC peptide and full-length α-synuclein have been reported to seed Aβ fibril formation *in vitro*^39^,^40^. Although other studies have failed to reproduce these *in vivo* findings^41^,^42^, we speculate that, *in vivo,* Aβ and α-synuclein could cross-react in processes that may be tuned by IDE binding.

In conclusion, our findings predict a twofold protective role for the chaperone-like activity of IDE *in vivo* related to interactions with α-synuclein; 1. IDE-α-synuclein interactions prevent the formation of α-synuclein amyloid fibrils and 2. the binding of α-synuclein to IDE enhances the proteolytical activity of IDE. Further studies are required to assess if the IDE-induced α-synuclein oligomers are arrested on- or off-path oligomers and if they are non-toxic, as well as what are contributions of other factors *in vivo*. Nevertheless, this study provides added support for a key role of IDE in antagonizing amyloid formation by neutralizing, either via dead-end binding or degradation mechanisms, amyloidogenic peptides.

## Methods

### Proteins

Recombinant IDE (Insulin-degrading enzyme, pET-His_1a) in Escherichia coli BL21 (DE3) RIL was expressed at 37 °C in LB media supplemented with 50 μg/mL kanamycin and grown until OD_600_ ~0.8. The cultures were shifted to 25 °C and IDE expression was induced with 0.5 mM IPTG, and the cells were grown for 6 h. The cells were centrifuged for 15 min at 5000 rpm, and the pellet re-suspended in re-suspending buffer (50 mM Tris, pH 8.0, 1 mM MgCl_2_) plus protease inhibitor and DNase-I, and sonicated on ice, followed by centrifugation at 20000 rpm for 40 mins. The supernatant was filtered through 0.2 μm syringe filter and loaded on an affinity column (Ni Sepharose 6 *HisPrep* FF 16/10, GE Healthcare), equilibrated with 50 mM Tris, 500 mM NaCl, 20 mM imidazole, 5%, pH 8.0. The column was washed overnight, and eluted with the same buffer containing 250 mM imidazole, and fractions containing IDE were collected. The pooled fractions were dialyzed against dialysis buffer (25 mM Tris, pH 8.0, 300 mM NaCl,10 mM imidazole) containing Tev-protease (ratio 1:100) at 4 °C. Tev-cleaved IDE sample containing Tev-protease was passed through the Ni Sepharose 6 *HisPrep* FF column, and the flow through containing IDE was collected. The sample was then loaded on an anion-exchange column (HiTrap MonoQ, GE Healthcare) equilibrated with 25 mM Tris, pH 8.0, 1 mM EDTA and eluted with a linear NaCl gradient of 25 mM Tris, 2 M NaCl, pH 8.0. Finally, the electrophoretically pure samples were pooled and concentrated, and run through a gel filtration column (Superdex S200 prep grade column, GE Healthcare), equilibrated with 25 mM Na-phosphate, pH 7.4, 150 mM NaCl. Concentration was determined using ε_280_ = 113,570 M^−1^ cm^−1^. Purified IDE was stored in 25 mM Na-phosphate, pH 7.4, 150 mM NaCl, at −80 °C. α-Synuclein in a pET-3a vector was transformed into BL21 (DE3) competent cells and grown at 37 °C in 5x LB medium supplemented with 100 mg/mL carbenicillin and grown until OD_600_ ~0.6. Protein expression was induced with 0.5 mM isopropyl b-D-1-thiogalactopyranoside, and the cells were further grown overnight. The cells were centrifuged for 30 min at 5000 rpm, and the pellet re-suspended in 8 M urea, 20 mM Tris, 20 mM imidazole, pH 8.0, and sonicated on ice, followed by centrifugation at 20000 rpm for 30 minutes. The supernatant was filtered and loaded on an affinity column (Ni Sepharose 6 Fast Flow, GE Healthcare), equilibrated with 20 mM Tris, 50 mM NaCl, 20 mM imidazole, 5% glycerol, pH 7.5, and eluted with the same buffer, but contained 250 mM imidazole. For removal of the His-tag as well as co-expressed protein, peptidase caspase 7 was added in a ratio of 1:100 (w/w), together with 20 mM 2-mercaptoethanol and was put to incubation over night at 4 °C. Cleavage efficiency was verified with SDS-PAGE, and selected fractions were diluted 1:1 (v/v) with Milli-Q water. The sample was then loaded on a anion-exchange column (HiTrap Q FF, GE Healthcare) equilibrated with 20 mM Tris pH 8.0, and eluted with a linear NaCl gradient of 20 mM Tris, 1 M NaCl, pH 8.0. Finally, α-synuclein was run through a gel filtration column (HiLoad 16/60 Superdex 75, GE Healthcare), equilibrated with 50 mM ammonium carbonate. The α-synuclein concentration was determined using the absorption at 280 nm.

### IDE protease activity assays

Mca-RPPGFSAFK(Dnp)-OH fluorogenic peptide substrate V from R&D Systems was used as substrate. IDE activity was measured for Serial dilutions of the internally quenched fluorogenic substrate-V, producing standard curves of enzyme activity. Enzyme activity (substrate cleavage) was measured in a fluorescence plate reader with excitation at 320 nm and emission at 405 nm in a Tecan Infinite M200 plate reader. To study the effect of α-synuclein or amylin presence, IDE activity was measured after pre-incubation of IDE with different concentrations of α-synuclein at 37 °C for 48 h, followed by adding fluorogenic substrate V and activity measurements.

### Protein fibrillization assays

Stock solutions were diluted to appropriate concentrations in 20 mM phosphate buffer at pH 7.4 containing 140 mM NaCl in a 96-well black plate. Aggregation experiments were performed at 37 °C with continuous agitation using a 2 mm glass bead in each well. All samples contained 20 μM thioflavin T (ThT); fluorescence was measured at 480 nm (excitation 440 nm) every 10 min in a Tecan Infinite M200 plate reader. The ThT assay was performed with 70 μM α-synuclein and varying concentrations of IDE (0–6 μM). Origin 8.0 was used to fit the data to standard sigmoidal equations. Graphs are representative of at least 3 independent experiments. Values are reported as normalized ThT fluorescence.

### Atomic force microscopy (AFM)

AFM measurements were performed on a BioScope Catalyst AFM (Bruker) in peak force mode in air at a resonance frequency of ca. 70 kHz and a resolution of 256 × 256 pixels. Samples were diluted to approximately 5 μM α-synuclein concentration with sterile filtered MilliQ water and applied to freshly cleaved mica surface (Ted Pella), incubated for 15 min, washed 3 times with MilliQ water and dried at room temperature.

### Fluorescent microscopy

Samples from completed ThT assays were diluted 1:10 and added to a glass slide and covered with a cover slip before being analyzed without delay in a Zeiss Imager Z1 (Zen software). Representative images display only the FITC channel. The same exposure times was used for all images.

### NMR

^1^H-^15^N NMR experiments were performed with a Bruker Avance III HD 850 MHz spectrometer equipped with a z-gradient cryoprobe using protein samples containing 5% D_2_O (v/v) in 20 mM phosphate buffer, 140 mM NaCl, pH 7.4 at 10 °C. The protein concentration was 40 μM α-synuclein and 40 μM IDE; the control of only α-synuclein had a concentration of 100 μM. The temperature was calibrated prior to the experiments by inserting a temperature probe into the sample compartment of the spectrometer. All data processing was made with NMRPipe^43^ and data were analyzed in NMRView^44^.

### ITC

Isothermal titration calorimetry (ITC) experiments were performed with an ITC_200_ (MicroCal) with either 200 μM α-synuclein monomers in the syringe and 20 μM IDE in the cell or 200 μM IDE in the syringe and 20 μM pre-formed α-synuclein oligomers (using the small molecule FN075^27^). In a typical run, 35 automated injections of 1.1 μl syringe protein with 300 s breaks in between injections were made at either 20 or 30 °C and 600 rpm stirring speed in low feedback mode. The cell and syringe samples were dialyzed against 20 mM phosphate buffer at pH 7.4 containing 140 mM NaCl prior to the experiments. Background heats of dilutions for appropriate samples injected to buffer were subtracted from each data set prior to analysis. Data integration, fitting and evaluation were performed using the software Origin™ 7 (ITC_200_ plugin from MicroCal).

### Size-Exclusion Chromatography (SEC)

Samples of IDE and α-synuclein (70 μM) were incubated at 37 °C for 48 h (20 mM phosphate buffer at pH 7.4 containing 140 mM NaCl) prior to elution on a Superose 6 gel filtration column (Amersham Pharmacia Biotech, GE Healthcare) at a flow rate of 0.5 mL/min. Selected fractions were analyzed by SDS-PAGE. To assess the monomer/dimer/tetramer status of IDE at different concentrations, samples with different starting IDE concentrations (5, 30 and 60 μM) were loaded on the Superose 6 gel filtration column and apparent molecular weights of the elution peaks were estimated from elution profiles of standard proteins (Bio-Rad). Concentrations of IDE in the elution peaks were determined using the extinction coefficient (dilution factor on this column is about 10 fold).

### Far-UV circular dichroism (CD)

CD spectra (190–320 nm) were recorded on Jasco 720 spectrophotometer (Jasco Corp., Tokyo, Japan) in a 1 mm cell. CD spectra of IDE alone (6 μM), α-synuclein alone (70 μM) and the mixture of IDE and α-synuclein were analyzed directly (10 min) and after 48 h of incubation (37 °C, agitation). Buffer signals were subtracted from all data.

### Dynamic light scattering (DLS)

Samples (same as for CD experiments) were analyzed on a Zetasizer Nano (Malvern, Malvern UK) probing scattered light at 173° using a 630 nm light source. 300 μl samples were analyzed in disposable cuvettes. Each sample was measured at least five times for 180 s. The autocorrelation curves were fitted with software provided by the instrument manufacturer using the Stokes–Einstein equation that assumes spheres. The samples were probed before and after 48 h incubation (37 °C, agitation).

## Additional Information

**How to cite this article**: Sharma, S. K. *et al.* Insulin-degrading enzyme prevents α-synuclein fibril formation in a nonproteolytical manner. *Sci. Rep.* **5**, 12531; doi: 10.1038/srep12531 (2015).

## Supplementary Material

Supplementary Information

## Figures and Tables

**Table 1:** Proteolytic activity data for IDE in the presence of α-synuclein.

	**Syn (μM)**	**k**_**cat**_ **(s**^**−1**^)	**K**_**M**_**(μM)**	**n**	**k**_**cat**_**/K**_**M**_**(M**^**−1**^**s**^**−1**^)
1 μM IDE	0	0.020 ± 0.001	20.1 ± 2.4	1.3 ± 0.1	1010
	1	0.024 ± 0.001	22.5 ± 1.6	1.4 ± 0.1	1070
	10	0.025 ± 0.002	21.6 ± 2.3	1.5 ± 0.2	1170
	100	0.058 ± 0.009	49.0 ± 13.2	1.2 ± 0.1	1190
	Syn (μM)	k_cat_ (s^−1^)	K_M_ (μM)	n	k_cat_/K_M_
3 μM IDE	0	0.241 ± 0.005	9.5 ± 0.4	1.9 ± 0.1	25400
	3	0.308 ± 0.010	11.2 ± 0.8	1.8 ± 0.2	27560
	35	0.397 ± 0.026	13.0 ± 1.6	1.6 ± 0.2	30490
	70	0.286 ± 0.009	10.0 ± 0.7	1.9 ± 0.2	28700
	Amylin (μM)	k_cat_ (s^−1^)	K_M_ (μM)	n	k_cat_/K_M_
1.6 μM IDE	0	0.082 ± 0.004	26 ± 2	1.3 ± 0.1	3100
	10	0.057 ± 0.006	41 ± 7	1.3 ± 0.1	1400

Enzymatic parameters (k_cat_, K_M_, n, k_cat_/K_M_) for IDE proteolytic activity on substrate V as a function of α-synuclein concentrations for two different IDE concentrations (1 and 3 μM) (data shown in Fig. 3). The initial rate versus substrate concentration data was fitted to an equation for a sigmoidal curve providing best fit values for k_cat_, K_M_, and n. k_cat_ is often referred to as the turn-over number and denotes the maximum number of enzymatic reactions catalyzed per second; K_M_ is an apparent dissociation constant that reflects the enzyme concentration where the rate of the enzyme reaction is half maximal; and finally, n reports on the cooperativity of the reaction with n = 1 lacking cooperativity. The ratio k_cat_/K_M_ corresponds to a pseudo-second order rate constant and reports on the catalytic efficiency. The variations in enzymatic parameters for 1 and 3 μM IDE as a function of α-synuclein are graphically visualized in Figure S7.
