# Supplementary material for: Insulin-degrading enzyme prevents α-synuclein fibril formation in a nonproteolytical manner
Source: Sci Rep. 2015 Jul 31;5:12531. doi: 10.1038/srep12531 (PMC4521159; doi:10.1038/srep12531)

Supplemental Information

Insulin-degrading enzyme prevents α-synuclein amyloid fibril formation in a nonproteolytical manner

Sandeep Sharma, Erik Chorell, Pär Steneberg, Emma Vernersson-Lindahl, Helena Edlund, Pernilla Wittung-Stafshede*

Content:

Figures S1-S7

Figure S1. A. Assessing possible ThT interaction with IDE. ThT fluorescence versus time (37°C, agitation) in the presence of various concentrations of IDE between 0-6.0 µM. **B**. Analyses of SDS-gel demonstrating Aβ (6 µg, AβCAM) degradation with time by IDE (1µg) on the left, and lack of α-synuclein (1.5 µg) degradation by IDE on the right. Proteins were incubated at time points indicated, up to 2 hours at 37°C in PBS buffer.

A.

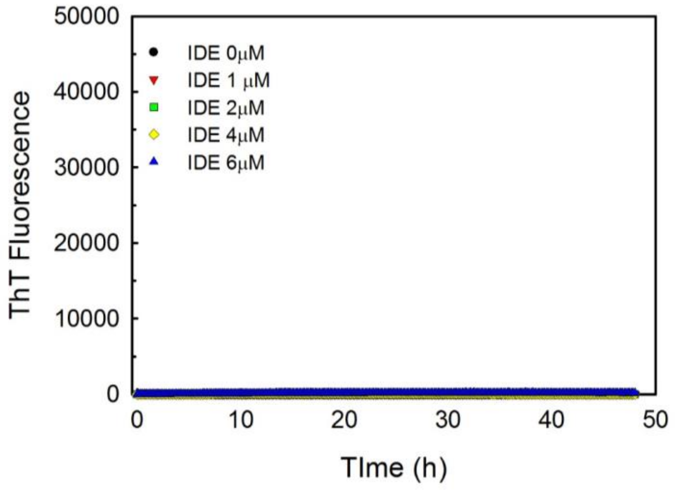

B

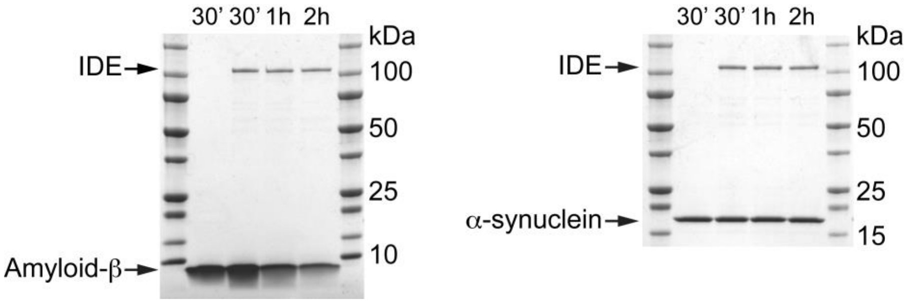

Figure S2. A. ThT fluorescence versus time (37°C, agitation) for 70 μM fresh α-synuclein in the presence of 1:1 and 1:0.5 molar ratios of BSA. (Separate experiments demonstrated that BSA alone did not enhance ThT emission.) **B.** ThT fluorescence versus time (37°C, agitation) for preformed amyloid fibers of 70 μM α-synuclein (48 h pre-incubation at 37°C, agitation), without and with the addition of 6 μM IDE to test for ability of IDE to dissolve preformed fibers.

A

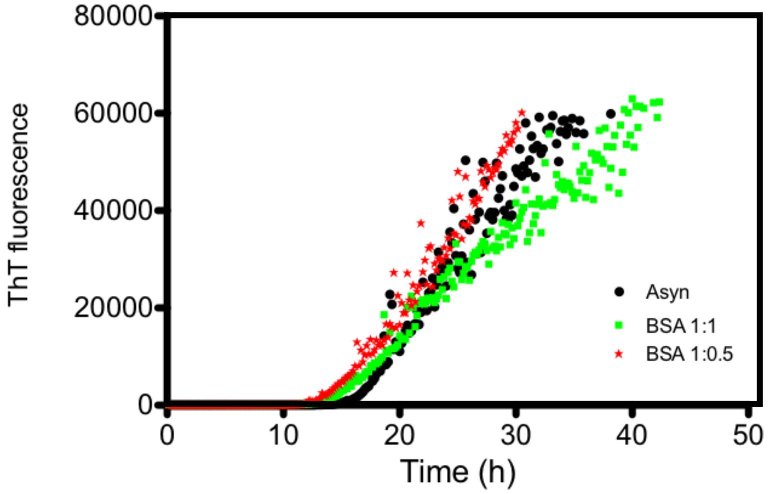

B

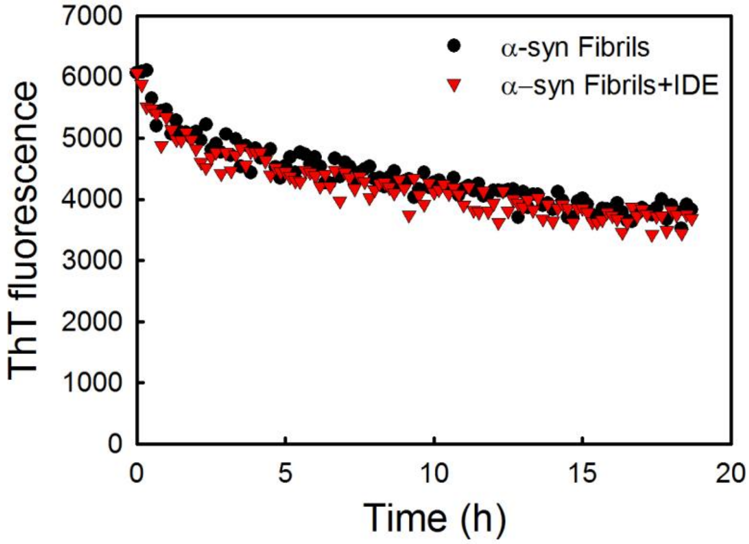

Figure S3. A. ThT fluorescence versus time (37°C, agitation) probing amyloid formation of 70 μM α-synculein in the presence of various concentrations of IDE, all with 2 mM ATP. The results are similar to the data without ATP. **B.** ThT signal after 48 h of incubation for each sample with (S3A) and without (from Figure 1A) ATP. Errors refer to triplicate experiments.

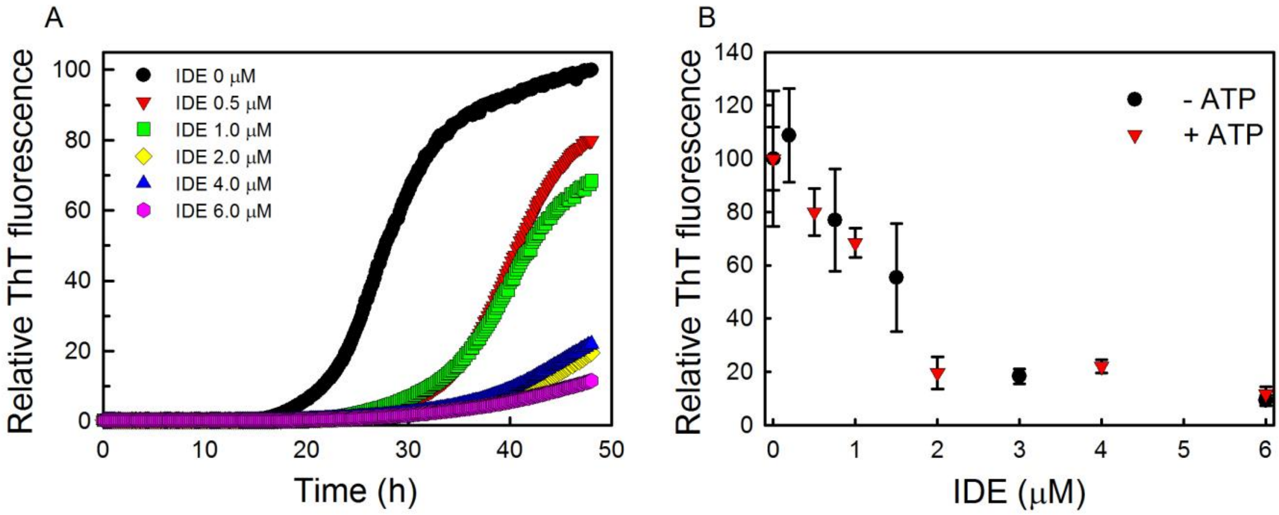

Figure S4. ITC thermograms of monomeric α-synuclein titrated to IDE at 20°C (**A**) and 30 °C (**B**) and IDE titrated to pre-formed α-synuclein oligomers at 20°C (**C**) and 30 °C (**D**). Background heats of dilution for titrations of the syringe samples into buffer in the cell have been subtracted. Because of the lack of any cooperative transition around 1 to 1 stoichiometry in the data in **A** and **B,** we conclude that IDE does not bind to α-synuclein monomers at these conditions. In contrast, because there is a cooperative transition with a negative enthalpy change at around a stoichiometric ratio of 0.2 (corresponding to roughly two-three IDE molecules per α-synuclein oligomer) in both **C** and **D**, IDE appears to bind to α-synuclein oligomers, although the low quality of the data prohibited determination of exact stoichiometry and thermodynamic parameters.

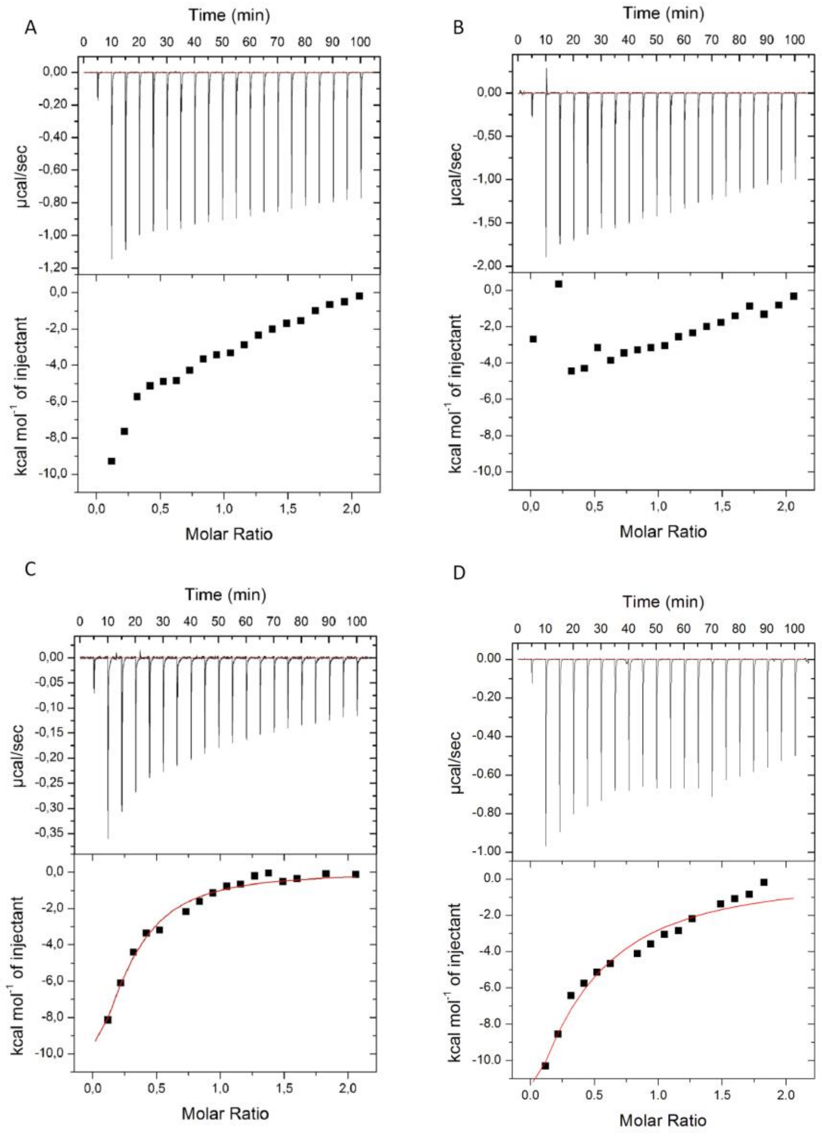

Figure S5. Characterization of IDE-α-synuclein oligomers.

A. Gel-filtration of an incubated IDE-synuclein mixture reveals the presence of oligomers eluting in the void of the column. Oligomers are known to decompose during SEC and, in accord, we also find synuclein and IDE individual fractions. SDS-PAGE analysis of the oligomer (void) fraction reveals the presence of the previously reported SDS-resistant 1:1 IDE:synuclein complex at 130 kDa. (Western blot analysis with appropriate antibodies, confirmed that both proteins were present in this fraction).The SEC data support that IDE binds to synuclein oligomers, but upon SDS-PAGE analysis only the 1:1 complex survives.

B. Far-UV CD spectra of 70 μM α-synuclein at time zero (monomers, unstructured according to CD) and at 48 h incubation (37°C, agitation; amyloid fibers based on AFM and ThT; β-structure according to CD) and of an IDE-synuclein mixture incubated for 48 h (37°C, agitation; oligomers according to AFM and DLS, β-structure according to CD). In the last CD spectrum, the contribution from IDE was subtracted (the CD signal of IDE did not change during the course of 48 h incubation).

C. DLS analysis (hydrodynamic radius, r_H, versus volume %) of α-synuclein, IDE and an IDE-α-synuclein mixture at 10 min after mixing (left) and after 24 h of incubation at 37°C with agitation (right). Synuclein alone has a small r_H at 0 h, indicative of monomers, whereas amyloid fibers are apparent at 24 h incubation (r_H approaching 1000 nm). IDE itself has a r_H of about 10-15 nm, in accord with a dimer at this condition. Incubation of synuclein in the presence of IDE results in assemblies with r_H of around 30 nm, in accord with oligomers.

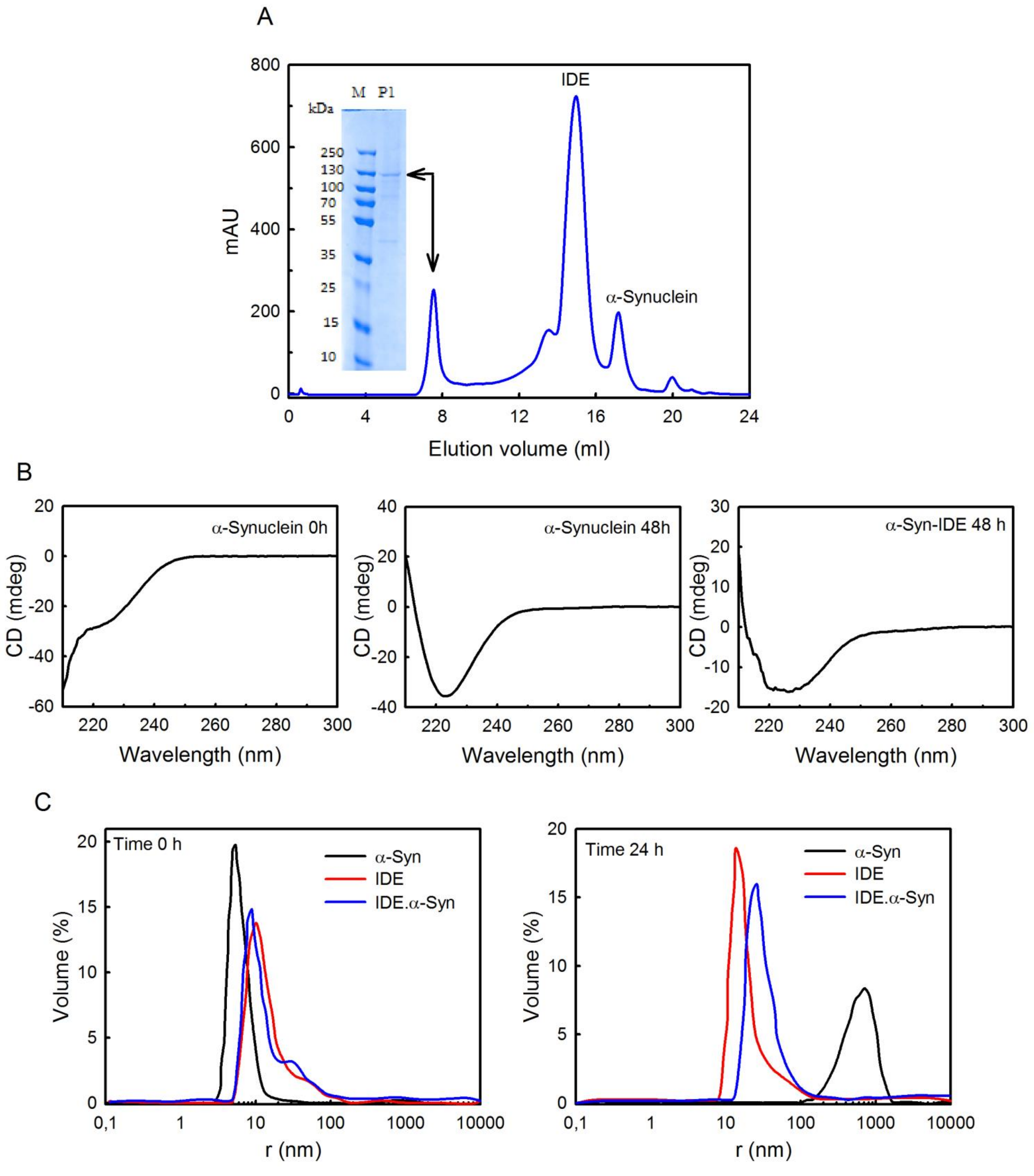
A
800
600
400
200
0
mAU
M P1
kDa
250
130
100
70
55
35
25
15
10
IDE
α-Synuclein
0 4 8 12 16 20 24
Elution volume (ml)
B
α-Synuclein 0h
α-Synuclein 48h
α-Syn-IDE 48 h
CD (mdeg)
Wavelength (nm)
220 240 260 280 300
C
Time 0 h
Time 24 h
α-Syn
IDE
IDE.α-Syn
Volume (%)
r (nm)
0,1 1 10 100 1000 10000

Figure S6. SEC elution profiles of IDE at different concentrations reveals a monomer-dimer(-tetramer) equilibrium. 5 μM (black), 30 μM (red) and 60 μM (blue) IDE samples were loaded on a Superose 6 gel filtration column and run at a flow rate of 0.5 mL/min. The dilution factor on the column is at least 10-fold. For the lowest IDE concentration, the protein elutes mostly as a monomer (peak I), whereas for the highest IDE concentration mostly dimers (peak II) are observed with the presence of a small fraction of tetramers (peak III). For the 30 μM IDE sample, the elution profile reveals both monomer and dimer peaks in almost equal amounts. In the latter, the dimer peak corresponds to an eluted concentration of 0.3 μM dimers and the monomer peak to an eluted concentration of 1 μM monomers and, from this, the estimated dimer-monomer dissociation constant is around 3 μM. Monomer, dimer and tetramer IDE peaks were assigned based on the elution profiles of protein standards.

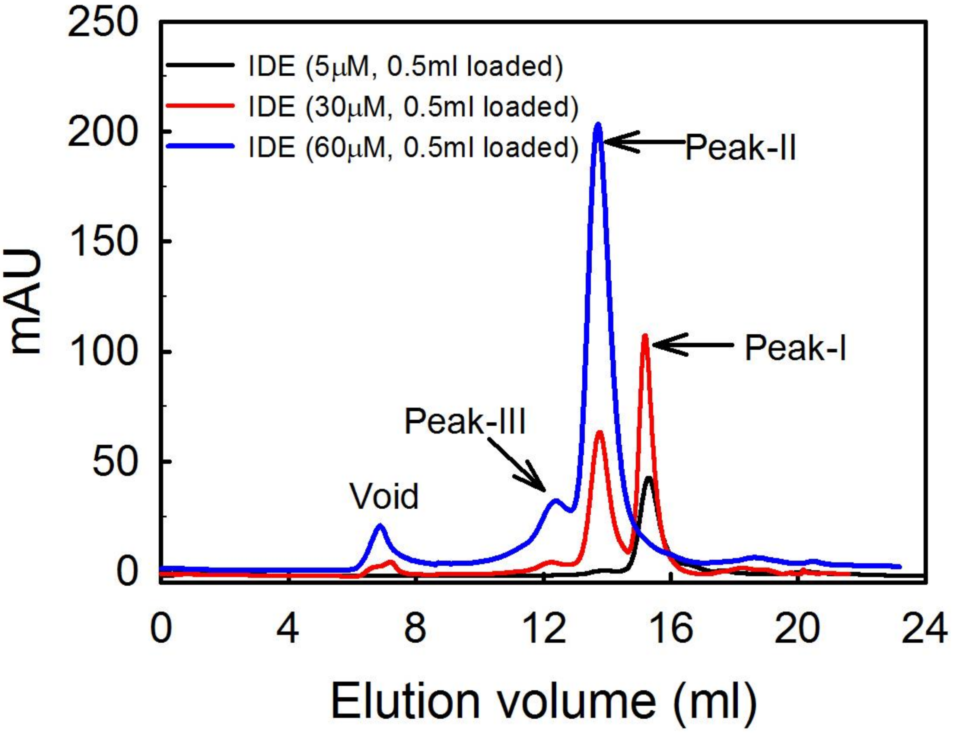

Figure S7. **Top two panels.** Variations in V_{max}, and V_{max}/K_M for 1 and 3 μM IDE activity as a function of α-synuclein concentration (data shown in **Figure 3AB**, values from **Table 1**). **Bottom panel.** Initial rates versus substrate concentration for IDE proteolysis of substrate V at 37°C, in the absence and presence of ThT (20 μM).

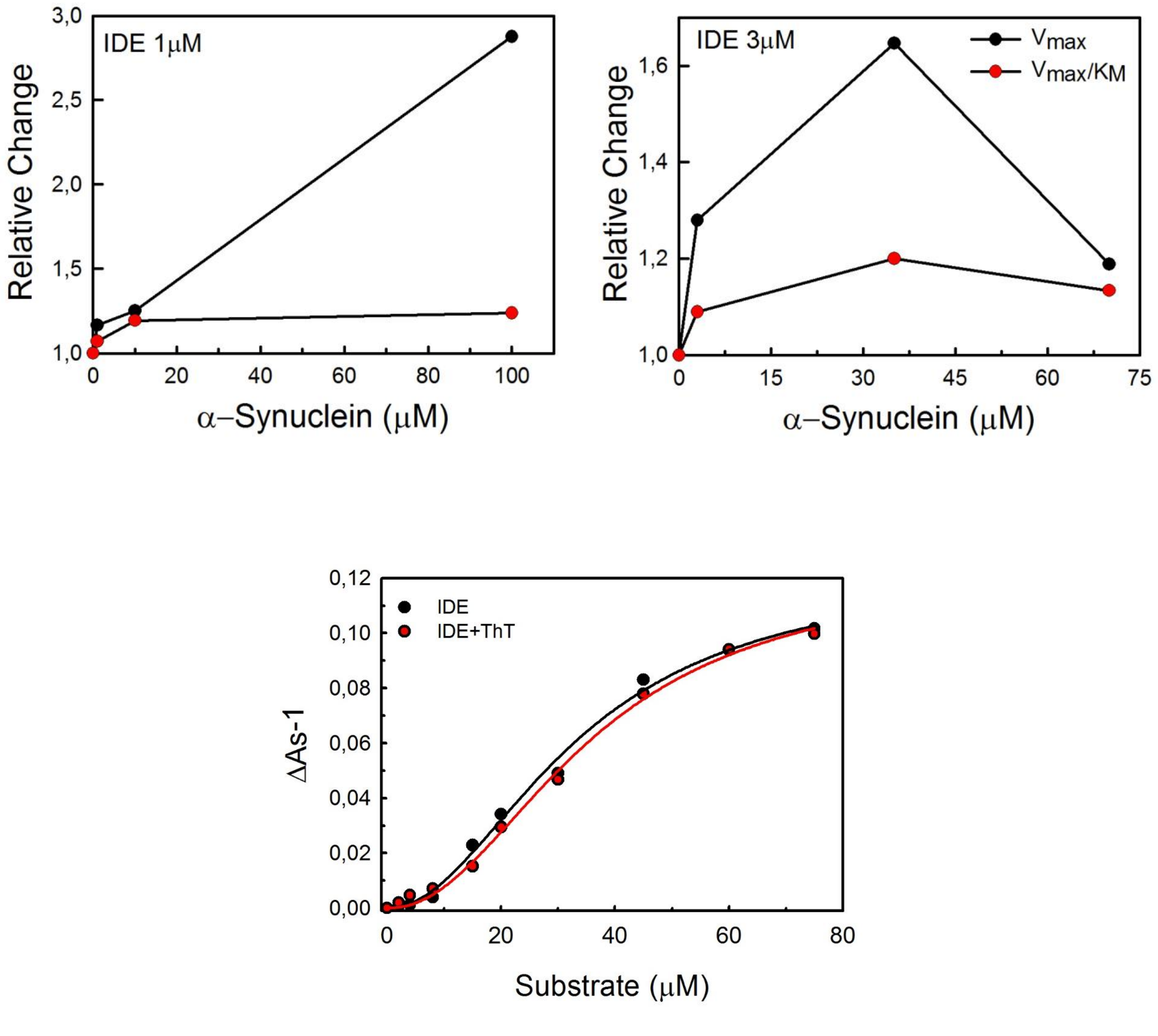

Supplement: Supplementary Information [file srep12531-s1.pdf]
